# The Government and the Hospitals

**Published:** 1921-07-02

**Authors:** 


### The Government and the Hospitals.

Sir Alfred Mond, the Minister of Health, has announced that the Government have decided, in view of the serious financial state of the voluntary hospitals, to ask the House of Commons to vote £500,000 in aid of the deficit during the present year. This money will be under the control of a Commission and Committees, as proposed by Lord Cave's Committee, who, it will be remembered, suggested that a sum of one million should be granted. The Government feel, however, that the present position of public affairs does not justify so large a contribution, and they hope that their action will stimulate voluntary effort and produce the balance required, thus maintaining the root principle of the voluntary system.

It is worth noticing in this connection that the Minister of Health for New South Wales has announced that his Government propose to divide New South Wales into 160 hospital districts, and to introduce legislation imposing on employer and employee the obligation of contributing regularly to the upkeep of the hospitals. The idea is that the Government will on their part pay a sum equal to the combined contributions. Voluntary contributions will still be encouraged for the purpose of expansion of buildings, and side by side with these the Government are prepared to pay one-third of such costs. We are afraid that this step will mean the eventual extinction of the voluntary system in New South Wales. The moral is that when State help comes in at the door voluntary support flies out at the window. Possibly the British Government had something of the kind in consideration when they decided to cut down the Cave requisition by half.

To put the hospitals right with the public we should like to see some acknowledgment made that the half-million is given as a special recognition of what the hospitals did in the way of extra service during the War. There is a precedent for this in the large grant made by Government to the Young Men's Christian Association which, under similar circumstances, found itself with a huge deficit at the end of the War. Without any fuss whatever the Government stepped in and footed the bill.

Government responsibilities have been supported by the voluntary hospitals in many ways during past years. In London alone, according to the Cave Report, more than £500,000 was spent during the War on Service patients in excess of what was provided under the capitation grant system. Surely for the whole country a million was little enough to ask in discharge of the debt.

